# Antifeedant Activity of Citrus Waste Wax and Its Fractions Against the Dry Wood Termite, *Cryptotermes* *brevis*

**DOI:** 10.1673/031.011.15901

**Published:** 2011-11-21

**Authors:** Ana Carolina Sbeghen-Loss, Mauricio Mato, Maria Veronica Cesio, Caren Frizzo, Neiva Monteiro de Barros, Horacio Heinzen

**Affiliations:** ^1^ University of Caxias do Sul, Biotechnology Institute, 95070-560, Caxias do Sul, RS-Brazil; ^2^ University of the Republic, Faculty of Chemistry, Box 1157, I 1800, Montevideo, Uruguay; ^3^ Aripê Citrus Ltda, BIO CITRUS, 95780-000, Montenegro, RS-Brazil

**Keywords:** bioactivity, coumarins, *Citrus latifolia* waste wax, wood protection

## Abstract

The wood protective action of citrus wax, a waste from the citrus industry that is a mixture of citrus fruit epicuticular waxes and essential oils, was evaluated against the termite *Cryptotermes brevis* Walker (Isoptera: Kalotermitidae). The antifeedant index (AI) of the total wax and fractions was calculated. The total citrus wax exhibited an AI_50_ value of 24.69 mg/cm^3^, the wax after hydrodistillation showed the strongest antifeedant property (AI_50_ 11.68 mg/cm^3^). Fractionation of the wax and gas chromatography—mass spectrometric analysis allowed the identification of coumarins and furancoumarins as the active compounds. These results suggest the potential use of these industrial residues as a natural approach to termite control.

## Introduction

Wood is widely used for a variety of purposes in both indoor and outdoor applications, but as any natural organic material, wood is susceptible to be degraded by many organisms (Yen and Chang 2008). Biodegradation of wood caused by termites is recognized as one of the most serious problems for wood utilization. In South America, there are at least six introduced termite species that have established themselves as urban pests (Constantino 2002). A few introduced *Cryptotermes* are the main structural pests of this group, particularly *Cryptotermes brevis* Walker (Isoptera: Kalotermitidae).

Chemical control is the preferred option worldwide to prevent termite attacks. Nevertheless, environmental pollution and health problems caused by the use of traditional wood preservatives or synthetic pesticides have increased the search for new and sustainable termite control methods. The use of pesticides from natural origins appears a particularly attractive alternative. Natural compounds are considered non—polluting agents, since they can be easily degraded and can be as effective as synthetic pesticides in the control of a wide array of pests (Chang et al 2001). Natural pesticides protect goods with different modes of action, by either killing the pest or acting as pest antifeedants or repellents. Although many natural sources of biopesticides have been described in the literature, their use has been scarce.

If a biopesticide is to be considered a viable alternative to the commonly used synthetic pesticides, it must have some key properties in addition to its biological activity; easily affordable, cheap, sustainably produced, and preferably with a known mode of action.

Thousands of tons of citrus wax, a waste from the citrus essential oil industry, is discarded every year; no useful application is described for it. In previous communications, we evaluated the toxic action of this unused raw material against *C. brevis*, a major wood pest (Sbeghen-Loss et al 2009). Their results showed that termites died at similar rates if they were either starved (controls) or fed on wood blocks treated with low concentrations of the wax or its fractions. Therefore, a possible antifeedant action can be postulated.

Antifeedants have been proposed as alternatives to synthetic insecticides (Ballesta-Acosta et al 2008). In the present communication we report the antifeedant action and chemical composition of citrus wax and their fractions against *C. brevis* to gather knowledge for the development of a new wood preservative.

## Materials and Methods

### General

All solvents were distilled from glass prior to use. SiO_2_ and thin layer chromatography (TLC) plates were from Machery-Nagel (www.mn-net.com). Gas chromatography— mass spectrometry (GC-MS) were run using a GC-17A Shimadzu gas Chromatograph (Shimadzu Corporation, www.shimadzu.com) equipped with a 25 m 0.25 mm HB-5 capillary column coupled to MS-5050 mass detector. The operating GC-MS conditions were: detector 280 °C, starting temperature 100 °C for 5 min, 10 °C/min to 280 °C for 25 min.

### Citrus Wax

Industrial wax samples of Bearss lime, *Citrus latifolia* Tanaka (Sapindales: Rutaceae), was collected from the semisolid precipitate resulting from the winterizing of citrus essential oils. The sample was supplied by Aripê Citrus Ltda, (Montenegro, RS, Brazil) and is hereby referred to as citrus waste wax (CWW).

### Essential oil distillation

The wax samples (31.7 g) were subjected to hydrodistillation in a Clevenger—type apparatus for two hours and the contents of the essential oil was studied by GC, GC-MS. Essential oil was stored in airtight containers prior the tests.

### Chemical fractionation/isolation

The aqueous phase yielded after hydrodistillation was extracted with ethyl acetate. The solvent was removed under reduced pressure and is hereby referred to as citrus fixed wax residue (CFWR). CFWR was fractionated through vacuum liquid chromatography (VLC) (Hostettman et al 1986) using Silica gel 60 (Merck, www.merck.com) as stationary phase, and Hexane/Ethyl Acetate (80:20), Hexane/Ethyl Acetate (50:50), and ethyl acetate as solvent to give three fractions named fraction A, B, and C respectively. The resulting fractions were concentrated under reduced pressure, separated by TLC and studied by GC, GC-MS.

### Melting point determination

250 mL of 2:1 n-propanol/water mixture in a 500 mL Bohemia glass were heated at a constant rate while being stirred with a magnetic rod. At 35 °C, 1 g of solid CWFR was added, and the heating continued while the temperature was recorded using a normal thermometer. The wax temperature melting range was recorded from the first droplet to a completely liquid drop (CWFR Mp: 75–79 °C).

### Termites

The test termite species *C.* *brevis* was collected in residences from Porto Alegre, RS, Brazil, and identified by Dr. Luiz Roberto Fontes (SP, Brazil). The colony was reared in the lab at 25 °C and 75% RH.

### Antifeedant bioassay

The antifeedant activity of CWW, the essential oil, CWFR, and the three resulting fractions from VLC (A, B, and C) were tested against pseudergates of *C. brevis* by using a non—choice assay as described by Simmonds et al (1990). Stock solutions in acetone were prepared. A series of five concentrations (5, 25, 50, 75, and 100 mg/cm^3^) of CWW and five concentrations (10, 20, 30, 40 and 50 mg/cm^3^) of essential oil, CWFR, and the three resulting fractions from VLC of each substrate were prepared by dilution with acetone. A series of five concentrations (5, 25, 50, 75, and 100 mg/cm^3^) of CWW were also evaluated after six months. *Pinus* sp. wood blocks measuring 2.54 × 2.54 × 0.64 cm were used. The blocks were dried in an oven at 103 ± 2 °C for four hours, and weighed individually to an accuracy of 0.1 mg. The wood blocks were then immersed in the acetone solutions of the tested extracts for 24 hours. Control wood blocks were immersed in acetone. After treatment they were placed in a laminar flow hood for 24 hours to eliminate the solvent and weighed to a constant weight. Five replicates of each concentration of the five solutions of the materials tested and a control solution were performed.

The termites were put in 9.1 cm diameter glass Petri dishes. After 60 days, dead and surviving termites were counted and the consumption of the wood blocks was evaluated. The wood blocks were weighed. The antifeedant indices (AI) were calculated following the methodology described by Simmonds et al (1990), according to the formula:

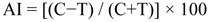

where C is the difference in weight (mg) between the control block before and after 60 days of bioassay and T is the difference in weight (mg) between the control block before and after 60 days of bioassay. The results were analyzed according Ballesta-Acosta et al (2008).

### Statistical analysis

The SPSS statistical program was employed to evaluate differences in the values for the antifeedant tests. Results with *p* < 0.05 were considered statistically significant. Probit analysis (Finney 1971) was used to determine the concentrations (EC_50_) with antifeedant effect (AI_50_). All results were obtained from independent experiments and expressed as mean ± SD.

### Chemical analysis

The materials under study were characterized through TLC using the following mobile phases; chloroform: chloroform-methanol 95:5, 90:1, and ethyl acetate:formic acid: acetic acid 100:11:11. The mobile phases and their major constituents were identified using GC-MS. The compounds having a > 90 Similarity Index (SI) in their EI mass spectrum with the matching compounds of the Wiley Registry of Mass Spectral Data (2000) were identified as such:

**7-Methoxycoumarin** MS, m/z, (%) [ ]: 176, (100), [M+]; 148, (80), [M^+^- CO]; 133, (90), [ M^+^- (CO + Me)]; 105, (15), [M^+^- (2CO + Me)].

**5,7-Dimethoxycoumarin** MS, m/z, (%) [ ]: 206, (100), [M^+^]; 178, (90), [M^+^- CO]; 163, (45), [M^+^- (CO + Me)]; 149, (8), [M^+^2CO]; 135, (25), [M^+^- (2CO+Me)]; 120, (10), [M^+^-(2CO+ 2Me)].

**5-Methoxypsoralen** MS, m/z, (%) [ ]: 216, (100), [M^+^]; 201, (35), [M^+^ - Me]; 188, (12), [M^+^- CO]; 173, (52), [M^+^- (CO + Me)]; 145, (25), [M^+^- (2CO+Me)].

**5,8- Dimethoxypsoralen** MS, m/z, (%) [ ]: 246, (100), [M^+^]; 231, (98), [M^+^ - Me]; 203, (23), [M^+^- (CO + Me)]; 188, (25), [M^+^(2Me + CO)]; 175, (35), [M^+^- (Me + 2CO)]; 160, (20), [M^+^- (2Me + 2CO)].

## Results and Discussion

### Chemical composition of CWW

The industrial wax employed was separated through hydrodistillation in two main fractions: the essential oil and a fixed wax residue (CWFR).

The essential oil of *C. latifolia* represented 34% of the CWW. The GC-MS analysis allowed the identification of twenty—seven constituents by comparing the obtained spectra with Wiley Registry (Wiley Registry of Mass Spectral Data 2000). The main components of the essential oil were limonene (59.56%), γ-Terpinene (13.27%), and βPinene (7.97%).

The fixed wax residue (CWFR) represented the other 66% of the original CWW. TLC and GC-MS analysis allowed its full characterization. The main components were wax hydrocarbons, waxy compounds, such as wax esters and fatty alcohols, and minor amounts (< 5%) of simple coumarins and furano coumarins. Four coumarins were identified: 7-methoxycoumarin, 5,7-dimethoxycoumarin, and the furancoumarins 5-methoxypsoralen and 5,8-dimethoxypsoralen. These compounds were also described previously in the peel of *C. latifolia* (Dugo et al. 1999). The fractionation through VLC did not succeed in separating the coumarins as a mixture of them was found in the three fractions. In fraction A, 7-methoxycoumarin, 5,7-dimethoxycoumarin, and two furano coumarins, 5-methoxypsoralen and 5,8-methoxypsoralen, were detected. The same four compounds were isolated in fraction B, but in different relative amounts. Fraction C was the only fraction that did not contain 7-methoxycoumarin.

**Table 1.**
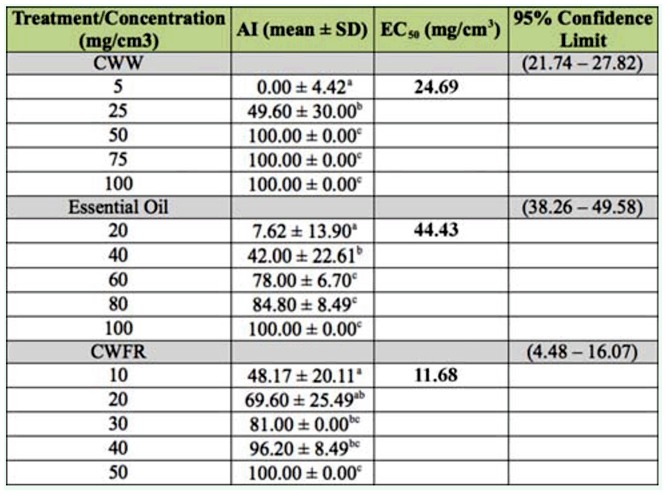
Antifeedant Index (AI) and Effective Concentration 50 (EC_50_) of citrus wax waste (CWW), the essential oil and the citrus fixed wax residue (CFWR).

### Antifeedant activity

The citrus wax waste (CWW), the essential oil, the wax after hydrodistillation (CWFR), and the three resulting fractions from VLC showed different levels of antifeedant activity when tested by a non—choice assay, using wood blocks that had been treated with the fractions under study against pseudergates of *C. brevis.* The antifeedant values are shown in Tables 1 and 2. The antifeedant activity of the assayed fractions was concentration—dependent in all cases.

The results showed that CWW and CWFR had the greatest antifeedant effect at 25 mg/cm^3^ and 10 mg/cm^3^ (AI = 100%) respectively, but none of the VLC fractions had high antifeedant activity. The most active was fraction C (AI = 57 in concentration at 20 mg/cm^3^) where the major compounds were 5,7-dimethoxycoumarin and 5-methoxypsoralen. The essential oil was less active, but at high concentrations it had statistically significant antitermitic properties.

**Table 2.**
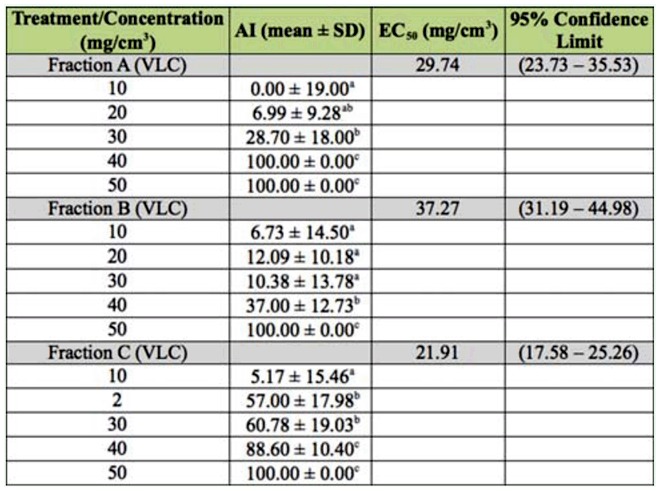
Antifeedant index (AI) and Effective Concentration 50 (EC_50_) of the VLC fractions from citrus fixed wax residue.

Tables 1 and 2 also shows the results of the probit analysis. Comparison with the EC_50_ values of the different fractions evaluated against C. *brevis* showed that fraction CWFR (EC_50_ = 11.68 mg/cm^3^) exhibited the highest antifeedant activity, followed by fraction C (EC_50_= 21.91 mg/cm^3^) and CWW (EC_50_ = 24.69 mg/cm^3^). The essential oil had less antifeedant effect (EC_50_ = 44 mg/cm^3^). The difference in antifeedant effects between CWW and CWFR is probably due to the dilution of the solid residue in the essential oil. None of the different relative amounts of coumarins present in each of the three VLC fractions were as effective as the coumarin proportion found in CWFR. The coumarins are a class of naturally occurring antifeedants. Calcagno et al. (2002) and Ballesta-Acosta et al. (2008) reported a deterrent effect of coumarins against *Spodoptera littoralis* with synergistic effects. According to Barros et al. (2008), the coumarins and furancoumarins present in the citrus waste wax had good insecticidal properties. The results shown above demonstrate that CWFR had a greater antifeedant effect compared to all the fractions studied. The simple separation procedure of the two main CWW groups of different volatility components adds value to an unused industrial waste. Additionally, the fixed residue is a very interesting raw material for antitermitic formulations. It has a relatively high melting point (75–79 °C) suitable for different industrial and household products. The wax can be used as is, or may also be included in commercial formulations that provide wood protection for six months as was demonstrated when EC_50_ was evaluated (EC_50_ = 7.35 mg/cm^3^).

The results obtained in this study show that citrus waste wax and some of its fractions have an interesting antifeedant effect against *C.* *brevis.* This unused waste from the citrus industry could be developed in a sustainable natural agent for termite control.

## Abbreviations:

**CFWR**,: citrus fixed wax residue;
**CWW**,: citrus waste wax;
**VLC**,: vacuum liquid chromatography
